# Supplementary material for: Pfizer-BioNTech (BNT162b2) Vaccine Effectiveness against Symptomatic Laboratory-Confirmed COVID-19 Infection among Outpatients in Sentinel Sites, Lebanon, July–December 2021
Source: Vaccines (Basel). 2024 Aug 23;12(9):954. doi: 10.3390/vaccines12090954 (PMC11436158; doi:10.3390/vaccines12090954)
Supplement: Supplementary file 1 [file vaccines-12-00954-s001.zip › S1 Table.pdf]

S1 Table: List of covariates affecting the likelihood of vaccination

**variable**	**Type**	**Values and coding**	**Definition**	**Source**
Smoking status				
Smoking status	Categorical	0. Not a smoker<br>1. Former smoker<br>2. Current smoker	if participant is a current smoker, or stopped since one year or not a smoker	interview
Nb of cigarettes/day	Numeric		Nb of cigarettes per day	interview
Nb of narguileh / week	Numeric		Nb of narguileh per week	interview
Nb of pipe	Numeric		Nb of pipe per week	interview
Health status				
Medical conditions	Categorical	0. No<br>1. yes	If participant was experiencing any medical conditions in the previous 12 months to testing	interview
List of chronic diseases: Diabetes , heart disease, hypertension	Categorical	1.yes, treated with medication<br>2.yes, not treated with medication<br>3. no<br>4. unknown	For each disease specify if treated or no with medication	Interview
Other health status	Text		Specify other health status, if not listed above	interview
Medical consultation	Categorical	1.yes<br>2.No	Visiting a physician for medical consultation in the	interview

		9. unknown	previous 12 months	
Number of medical consultations	Numeric		Average number of visits	interview
Hospital admission	Categorical	1.yes<br>2.No<br>9. unknown	Admission to hospital in the previous 12 months	interview
Number of hospital admissions	Numeric		Average number of hospital admission	interview
Living arrangements				
Number of households members	Numeric		Specify number of households members living with participant	interview
Numbers of rooms	Numeric		Specify number of rooms at home (excluding kitchen	interview
Basic needs coverage	Categorical	1. I can cover all my basic needs<br>2. I can cover my basic needs with difficulties<br>3. I can cover partially<br>4. I can't cover	Basic needs coverage	interview
Income origin	Categorical	1. Work income<br>2. retirement payment<br>3.family help<br>4.financial help not	Origin of income	interview

		family related<br>5. help from abroad<br>6. personal savings<br>7. no income<br>8. refuse to answer<br>9. Other		
Exposure to SARS-COV-2 in the community				
Contact with a confirmed case	Categorical	0.No<br>1.yes	If participant has a contact with confirmed case in the past 14 days prior to testing	interview
Public transportation	Categorical	1.Always<br>2.Sometimes<br>3.Never<br>4.Not applicable	If participant used public transportation in the previous 6 months to testing	interview
Attending social events	Categorical	1.Always<br>2.Sometimes<br>3.Never<br>4.Not applicable	If participant attended social events in the previous 6 months to testing	interview
Travel	Categorical	1.Always<br>2.Sometimes<br>3.Never<br>4.Not applicable	If participant traveled in the previous 6 months to testing	interview
Adherence to NPI				
Use of mask	Categorical	1.Always	If participant used masks outdoor in	interview

		2.Sometimes<br>3.Never<br>4.Not applicable	the previous 6 months to testing	
Social distancing	Categorical	1.Always<br>2.Sometimes<br>3.Never<br>4.Not applicable	If participant ensure social distancing in the previous 6 months to testing	interview
Wash hands with soap and water	Categorical	1.Always<br>2.Sometimes<br>3.Never<br>4.Not applicable	If participant use soap and water in the previous 6 months to testing	interview
Use of hand sanitizer	Categorical	1.Always<br>2.Sometimes<br>3.Never<br>4.Not applicable	If participant use hand sanitizer in the previous 6 months to testing	interview
