# Supplementary material for: Pfizer-BioNTech (BNT162b2) Vaccine Effectiveness against Symptomatic Laboratory-Confirmed COVID-19 Infection among Outpatients in Sentinel Sites, Lebanon, July–December 2021
Source: Vaccines (Basel). 2024 Aug 23;12(9):954. doi: 10.3390/vaccines12090954 (PMC11436158; doi:10.3390/vaccines12090954)
Supplement: Supplementary file 1 [file vaccines-12-00954-s001.zip › S2 Table.pdf]

S2 Table : Behavioural factors associated with COVID-19 diagnosis, July-December 2021, Lebanon (n=457)

Variables		Cases (n=150)		Controls (n=307)		p-value
		Number	%	Number	%	
**Contact with confirmed or suspected COVID-19 case in the past 14 days**	Yes	57	39	87	29	0.022
	No	88	61	218	71	
**Exposure to SARS-CoV-2 in the community in the previous 6 months**						
**Using public transportation**	Always	22	15	64	21	0.137
	Sometimes	40	28	95	31	
	Never	83	57	145	48	
**Attending social events**	Always	18	13	25	8	0.237
	Sometimes	67	46	133	44	
	Never	60	41	146	48	
**Traveling**	Always	4	3	3	1	0.134
	Sometimes	5	3	21	7	
	Never	135	94	280	92	
**practice of non-pharmaceutical interventions (NPI) in the past 6 months**						
**Using mask when going outdoor**	Always	100	69	194	64	0.283
	Sometimes-never	45	31	110	36	
